# Evaluation of AI‐based auto‐contouring tools in radiotherapy: A single‐institution study

**DOI:** 10.1002/acm2.14620

**Published:** 2025-01-21

**Authors:** Tingyu Wang, James Tam, Thomas Chum, Cyril Tai, Deborah C. Marshall, Michael Buckstein, Jerry Liu, Sheryl Green, Robert D. Stewart, Tian Liu, Ming Chao

**Affiliations:** ^1^ Department of Radiation Oncology Icahn School of Medicine at Mount Sinai New York New York USA; ^2^ Department of Radiation Oncology The Mount Sinai Hospital New York New York USA; ^3^ Department of Radiation Oncology Mount Sinai Union Square New York New York USA; ^4^ Department of Population Health Science and Policy Icahn School of Medicine at Mount Sinai New York New York USA

**Keywords:** artificial intelligence, image segmentation, organ at risk, Radiation therapy, treatment planning

## Abstract

**Background:**

Accurate delineation of organs at risk (OARs) is crucial yet time‐consuming in the radiotherapy treatment planning workflow. Modern artificial intelligence (AI) technologies had made automation of OAR contouring feasible. This report details a single institution's experience in evaluating two commercial auto‐contouring software tools and making well‐informed decisions about their clinical adoption.

**Methods:**

A cohort of 36 patients previously treated at our institution were selected for the software performance assessment. Fifty‐eight OAR structures from seven disease sites were automatically segmented with each tool. Five radiation oncologists with different specialties qualitatively scored the automatic OAR contours’ clinical usability by a 4‐level scale (0–3), termed as quality score (QS), representing from “0: not usable” to “3: directly usable for a clinic.” Additionally, quantitative comparison with clinically approved contours using Dice similarity coefficient (DSC) and the 95% Hausdorff distance (HD95) was performed in complement to QS from physicians.

**Result:**

Software A achieved an average QS of 2.17 ± 0.69, comparable to Software B's average QS of 2.17 ± 0.72. Software B performed better with more OARs (42 vs. 37) that required minor or no modification than Software A. Major modifications were needed for 13 out of 58 automated contours from both tools. Both DSC and HD95 scores for the two tools were comparable to each other, with DSC: 0.67 ± 0.23 versus 0.66 ± 0.21 and HD95: 13.07 ± 15.84 versus 15.55 ± 18.45 for Software A and Software B, respectively. Correlation coefficients between the physician score and the quantitative metrics suggested that the contouring results from Software A aligned more closely with the physician's evaluations.

**Conclusion:**

Based on our study, either software tool could produce clinically acceptable contours for about 65% of the OAR structures. However, further refinement is necessary for several challenging OARs to improve model performance.

## 1 INTRODUCTION

Technological advances have prompted the evolution of radiation therapy into a precise treatment modality. Sophisticated techniques such as intensity‐modulated radiation therapy (IMRT) and/or proton therapy have been routinely employed to improve the target delivery of radiation dosage and better protect the surrounding tissues using steep dose gradients, entailing accurate delineation of the organs at risk (OARs). Traditionally, this step in the treatment planning is performed manually, which is however labor‐intensive and operator‐dependent. Hence, automated contouring was introduced to mitigate these issues. In the pre‐artificial intelligence (AI) era, this was achieved with the widely used atlas‐based segmentation because of the consistency between anatomical structures. The atlas‐based methods utilize registration techniques to solve the segmentation problem and are robust to image noise, but they often lack the flexibility in locally fine‐tuning the object boundaries, leading to poor accuracy.^1^, ^2^, ^3^ The other family of conventional segmentation approaches is based on the active contours which are driven by a pre‐defined energy function in a varying manner and are capable of capturing the local shape features. Nevertheless, these methods are sensitive to the initialization and the shape of the structure to be segmented may not be well preserved. Additionally, the pre‐defined energy functional may not well represent the structure's characteristics.^3^, ^4^ These drawbacks make the conventional approaches less robust and thus impractical in daily applications.

AI, particularly deep learning based on the convolutional neural network and the most recent transformer models, has shown great success in the computer vision and biomedical domains. It has also spurred a wave of creating new tools for automatic contouring in the field of radiation therapy. Compared to the conventional segmentation approaches, AI‐based tools offer many potential advantages in terms of accuracy, consistency, and quality. Besides the innovative research being actively explored by academic researchers, there are many commercial products available from established vendors and startup companies. These efforts, particularly the commercially available tools, have enabled radiation therapy clinics to substantially improve the OAR delineation efficiency as well as reduce the inter‐observer variations in manual segmentation.

While the availability of numerous products is beneficial, it poses a challenge for users to determine which product to select. Although there have been publications assessing and comparing automatic contouring tools,^5^, ^6^ we find it essential to share our experience in evaluating automated contouring tools from two major companies, referred to as software A and software B in this report. Our decision to omit product names was made to focus on the methodology rather than publicly endorsing or comparing specific products. Additionally, there are legal and institutional constraints that prevent us from disclosing the product names. Our findings from this evaluation process complement existing studies and could aid other clinics in their product selection. Throughout this report, the terms contouring, delineation, and segmentation are used interchangeably.

## 2 METHODS

### 2.1 Clinical data collection

We focused on seven major disease sites: brain, breast, male and female pelvis, lung, head and neck, and abdomen. For the software assessment, we selected a cohort of 36 patients previously treated at our institution. This cohort included six breast cancer patients and five patients for each of the remaining six sites. The planning computed tomography (CT) images for these patients were meticulously chosen to ensure the absence of significant artifacts and provide a fair representation of the patient population regarding patient anatomical variations.

### 2.2 Manual contouring

The existing OAR contours in the Eclipse treatment planning system (Varian Medical Systems, Palo Alto, California, USA), delineated by planners and approved for treatment by radiation oncologists, were used as reference or “manual” contours for the quantitative software assessment. Although inter‐observer variability of contouring exists, as demonstrated by a previous study,^7^ we ensured that all the chosen OARs were contoured in accordance with the Radiation Therapy Oncology Group (RTOG) guidelines. Consequently, these contours could reasonably serve as the ground truth, although there are several caveats with this choice, which are discussed later in the Discussion section.

### 2.3 Commercial software A and B

Although the names of these products are not provided in this article, relevant information about these two products including the model training and datasets used to build the models is useful to the readers. The specific algorithms and architectures used in both products are proprietary and not fully disclosed to us, but we learned that both deep learning models were based on the U‐Net architecture and were pre‐trained using publicly available CT and MRI scans, for example, The Cancer Imaging Archive (TCIA) and the Cancer Genome Atlas Program (TCGA) collections. For Software B, they employed CT images from two institutions for their model training and validation in addition. Our decision of selecting these two products were made after reviewing the available products on the market, and, more importantly, consulting with other institutions who underwent similar processes and learning from their experiences.

Software A was installed as a standalone application which is not attached to the treatment planning system. Once initiated, it automatically reads in CT images that were exported from CT simulation. Users would need to assign contour models according to the specific disease site. The OARs to be segmented are often pre‐configured in the model thus only a single click is needed to start the contouring process. After the contouring is complete, the file containing the generated OAR contours can be imported together with the original CT images into treatment planning system for the subsequent planning work.

Software B, a well‐integrated cloud‐based automated contouring solution, was installed as a script built‐in to the Eclipse treatment planning system. Software B identifies the newly imported CT images, generates all OAR contours, and assigns them to individual structures. Users can manually adjust the assignment to ensure that all available structures are assigned correctly with the generated contours and need to review and approve each structure by scrolling over all slices of the contour before they can be exported back to the treatment planning system. If no adequate match is found or the user does not approve the contour, an empty structure will be returned.

### 2.4 List of contours

Table 1 summarizes the delineation status of the OAR structures that were selected for the software assessment. The structure names are listed in the first column and the frequency of each OAR in the patient cohort is shown in the second column. The status for the contours generated manually, automatically by software A and B are described in the third through fifth columns, respectively, where Y stands for the status that the contour was successfully generated, and N stands for that not generated.

**TABLE 1:** Delineation status of the OAR structures selected for software assessment.

Structure	No. of pt.	Manually	Software A	Software B
Bowel bag	20	Y	Y	Y
Bladder	10	Y	Y	Y
Mandible bone	5	Y	Y	Y
Bowel	15	Y	Y	Y
Left brachial plexus	10	Y	Y	Y
Right brachial plexus	10	Y	Y	Y
Brain	10	Y	Y	Y
Brainstem	10	Y	Y	Y
Left breast	5	Y	N	Y
Right breast	5	Y	N	Y
Bronchus	5	Y	Y	Y
Cauda equina	15	Y	Y	Y
Oral cavity	5	Y	Y	Y
Left chest wall	5	N	Y	Y
Right chest wall	5	Y	Y	Y
Left cochlea	10	Y	Y	Y
Right cochlea	10	Y	Y	Y
Duodenum	5	N	Y	Y
Esophagus	10	Y	Y	Y
Left eye	10	Y	Y	Y
Right eye	10	Y	Y	Y
Left femoral head	10	Y	Y	Y
Right femoral head	10	Y	Y	Y
Left lacrimal gland	5	Y	Y	Y
Right lacrimal gland	5	Y	Y	Y
Left submandibular gland	5	Y	Y	Y
Right submandibular gland	5	Y	Y	Y
Heart	5	Y	Y	Y
Left hippocampus	5	N	Y	Y
Right hippocampus	5	N	Y	Y
Left kidney	5	Y	Y	Y
Right kidney	5	Y	Y	Y
Larynx	5	Y	Y	Y
Left lens	10	Y	Y	Y
Right lens	10	Y	Y	Y
Lips	5	Y	Y	Y
Liver	10	Y	Y	Y
Pelvic lymph nodes	10	Y	Y	Y
Left lung	5	Y	Y	Y
Right lung	5	Y	Y	Y
Constrictor muscle	5	Y	Y	Y
Optic chiasm	10	Y	Y	Y
Left optic nerve	10	Y	Y	Y
Right optic nerve	10	Y	Y	Y
Pancreas	5	Y	N	N
Left parotid	5	Y	Y	Y
Right parotid	5	Y	Y	Y
Penile bulb	5	Y	Y	Y
Pituitary	5	N	N	Y
Prostate	5	Y	Y	Y
Rectum	10	Y	Y	Y
Seminal vesicles	5	Y	Y	Y
Spinal cord	15	Y	Y	Y
Stomach	10	Y	Y	Y
Trachea	5	N	Y	Y
Uterus cervix	5	N	Y	Y
Vagina/Genitalia	5	Y	N	Y
Left ventricle	5	Y	Y	N

*Note*: “Y” indicates the contour was generated, while “N” indicates it was not generated.

### 2.5 Comparison of contouring quality

The pivotal criterion, when evaluating the performance of automatic tools, is the contouring accuracy although other factors such as speed and convenience are also important. For that purpose, we adopted two sets of metrics: qualitative and quantitative. The former is based on radiation oncologists’ opinions and the latter is on the statistical comparison between the automated contours and the previously approved ones used for treatment planning.

### 2.6 Qualitative metric

When reviewing the automated contours for their clinical usability, the participating radiation oncologists provided a quality score (QS) revealing how much work is needed to modify them so that clinically acceptable accuracy can be achieved. This metric, termed as the “degree of editing,” is a commonly used subjective evaluation metric system^8^, ^9^, ^10^ for each automated contour according to the criteria stipulated in Table 2.

**TABLE 2:** Qualitative scoring criterion for the clinical usability of the automated contours.

Quality Score	Meaning (Degree of editing needed to clinical acceptance)
0	Not usable, erase all and re‐contour manually will be faster.
1	Major modification is needed, still usable and saves a bit time.
2	Minor modification is needed, usable and saves a lot of time by quick modifications.
3	Perfect! Ready for use as is in the clinically acceptable level. No modification is necessary.

### 2.7 Quantitative metric

Two quantitative metrics were calculated to assess the quality of automated contours: Dice similarity coefficient (DSC), and HD95, the 95% percentile of the maximum distances of the automated contours to the nearest point in the reference ground truth contours.^11^, ^12^

DSC has been widely used in evaluating image segmentation and other contour comparison studies. It is defined as

$$\begin{array}{c} \mathrm{DSC}=\frac{2*\left|A\;\cap \;B\right|}{\left|A\right|+\left|B\right|}\;\left(1\right) \end{array}$$

where A and B represent the volumes of two structures, respectively. DSC approaches 1.0 when the two volumes overlap exactly with each other, indicating a perfect match with the original manual contour. DSC, however, is sensitive to the structure's absolute volume as small structures can be over penalized, while large structures may have their overlap over‐exaggerated. To eliminate the impact of a very small subset of the outliers and decrease potential bias, HD95 is used together with DSC for more objective evaluation by focusing more on the shape and edge outlines of the structure contours rather than the overlapping volume ratios. By definition, the HD between the two sets is the maximum distance of a point in one set to its nearest point in the other set. HD95 in this study thus represents the maximum distance between the two contours such that 95% of the surface points of reference contours are within this distance from the automated contours. In the following formula,

$$\begin{array}{c} d_{H}\;\left(A,B\right)==max\left\{sup_{a\in A}inf_{b\in B}d\left(a,b\right),\right.\\ \left.sup_{b\in B}inf_{a\in A}d\left(b,a\right)\right\}\left(2\right) \end{array}$$

where d(a,b) is the distance between points a and b; sup denotes the supremum (least upper bound) of a set and inf denotes the infimum (greatest lower bound) of a set. HD95 is calculated bidirectionally and, hence, if the boundary is outside the original structures, it is positive and vice versa. The calculation is extended to 3D by using binary masks. A close‐to‐zero HD95 indicates a more similar contoured structure.

It is useful to check the consistency between subjective QS and each of the two quantitative metrics DSC and HD95. This is done through calculating the correlation coefficient between them using the following formula:

$$\begin{array}{c} \mathrm{Correlation}\;\left(X,Y\right)=\frac{\sum (x-\bar{x)}(y-\bar{y)}}{\sqrt{\sum (x-{\bar{x)}}^{2}\sum (y-{\bar{y)}}^{2}}}\;\left(3\right) \end{array}$$

In this study, the strength of the association is labeled by the absolute value of the correlation coefficients: 0–0.19 is regarded as “very weak,” 0.2–0.39 as “weak,” 0.40–0.59 as “moderate,” 0.6–0.79 as “strong,” and 0.80–1 as “very strong” correlation.

## 3 RESULTS

**FIGURE 1:**
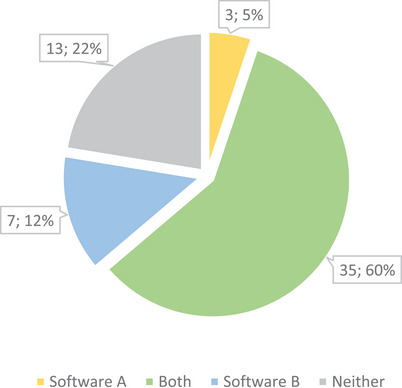
Overview of the contouring performance of the two software tools on 58 OAR structures based on the quality score > 2.

The pie chart in Figure 1 represents the number of OAR structures evaluated with the criterion of QS > 2 (a quality that minimal modification is needed) for the two software tools. According to the physician score, both tools achieved acceptable accuracy (QS > 2) for 35 out of 58 OAR structures, representing 60% of the total. Software A could accurately delineate three (5%) additional OARS, while Software B could delineate seven (12%) more, based on QS > 2. The remaining 13 OAR structures (22%) contoured by both tools had a QS < 2. To further assess global performance, we calculated the average scores for each of the three metric sets using all OAR contours and the results are summarized in Table 3. No significant differences were observed between the two software tools, regardless of the evaluation metric.

**TABLE 3:** Comparing average scores in each metric for automated contour assessment of software A and Software B.

	Quality scores	DSC	HD95
Software A	2.17 ± 0.69	0.67 ± 0.23	13.07 ± 15.84
Software B	2.17 ± 0.72	0.66 ± 0.21	15.55 ± 18.46

**FIGURE 2:**
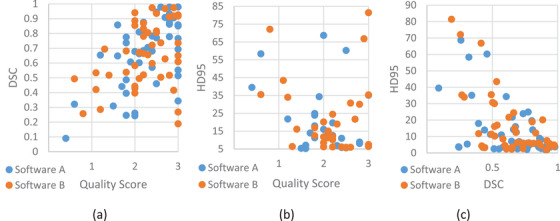
Scatter plots showing the software performance with qualitative and quantitative metrics. (a) Quality score vs. DSC; (b) Quality score vs. HD95; (c) DSC vs. HD95. Software A's scores are labeled in blue and Software B's scores in orange.

Figure 2 illustrates software performance through three scatter plots based on both qualitative and quantitative metrics. The first two scatter plots display the relationship between the qualitative score (QS) on the horizontal axis and the quantitative metrics DSC (Figure 2a) and HD95 (Figure 2b) on the vertical axis for each of the 58 OAR contours. Figure 2c shows the scatter plot of HD95 against DSC. In these plots, blue dots represent scores from Software A, while orange dots represent scores from Software B. Fitting a boundary circle to the points reveals a pattern indicating where well‐contoured OARs are located. Specifically, automatically generated OAR contours that meet the criteria of QS > 2 and DSC > 0.7 are found in the upper right corner of Figure 2a, with 39 OAR contours considered to be well delineated by Software A and 35 by Software B. For Figure 2b, contours meeting QS > 2 and HD95 < 20 are located in the lower right corner, with 32 contours from Software A and 29 from Software B satisfying this criterion. Finally, in Figure 2c, with the criterion of DSC > 0.7 and HD95 < 20, the numbers of well‐delineated contours are 21 for Software A and 20 for Software B, respectively.

The correlation coefficients between the subjective score QS and each of the two objective scores DSC and HD95 (shown in columns 2 and 3), as well as between DSC and HD95 (in the last column), are summarized in Table 4. The correlation between QS and both DSC and HD95 is stronger for Software A than for Software B, suggesting that the contouring results from Software A align more closely with the physician's evaluations. For both software tools, the correlation between DSC and HD95 is stronger than that between QS and either DSC or HD95, which is as expected since DSC and HD95 are supposed to be strongly correlated. However, the coefficients between DSC and HD95 are 0.53 and 0.63 from Software A and B, indicating a moderate correlation. This reflects that DSC and HD95 represent different aspects of the consistency between the automated contours and the reference contours, which are delineated differently, as discussed later.

**TABLE 4:** Correlation coefficients between quality score and each of the quantitative metrics, DSC, and HD95, as well as between DSC and HD95 for two software products.

	Quality score vs. DSC	Quality score vs. HD95	DSC vs. HD95
Software A	0.58	0.47	0.53
Software B	0.34	0.21	0.63

**FIGURE 3:**
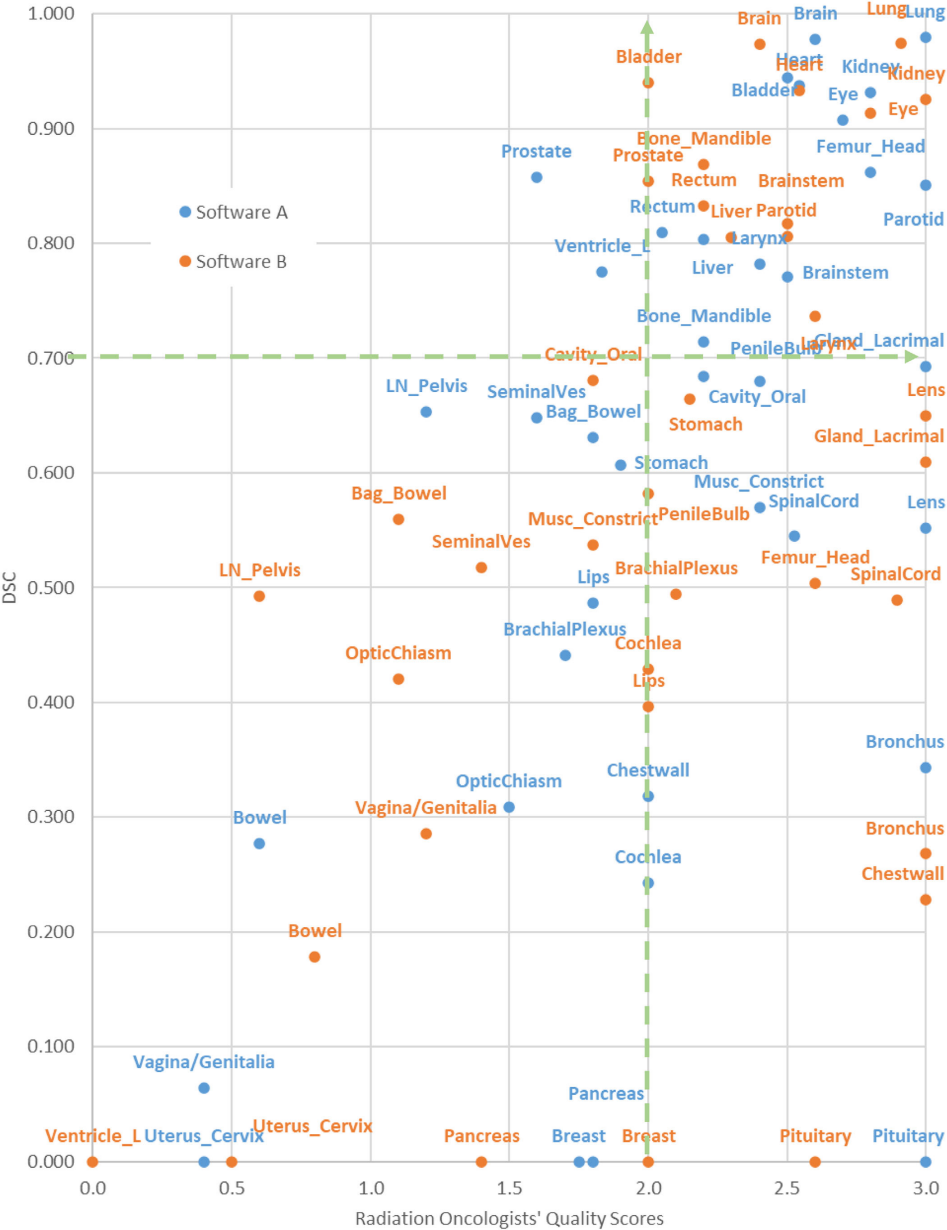
Average quality scores vs. DSC for automated OAR contour structures divided into four quadrants.

Utilizing both qualitative and quantitative metrics, we divided the chart of QS versus DSC using the metric of QS = 2 and DSC = 0.7 into four quadrants for better visual inspection and analysis. For example, to visualize the quadrants across the majority of the OAR structures, we plotted an enlarged view of Figure 2a in Figure 3. Thirty‐seven out of 58 OARs were randomly selected for better visualization. The full classification for OAR contours in each quadrant is presented in Table 5. In the first quadrant, there are 21 OAR structures from Software A and 20 from Software B. Among them, 18 OAR contours were generated by both tools These OARs include bladder, mandible bone, brain, brainstem, both eyes, right lacrimal gland, left submandibular gland, heart, kidneys, larynx, liver, lungs, parotid, and rectum. Additionally, Software A segmented both femoral heads and the right submandibular gland with high accuracy, whereas Software B performed better with the prostate gland and right breast.

**TABLE 5:** Summary of OAR structures in each quadrant on the QS vs. DSC plot.

Quadrant 1 DSC > 0.7 QS > 2	Common (18)	Bladder, Mandible Bone, Brain, Brainstem, Left Eye, Right Eye, Right Lacrimal Gland, Left Submandibular Gland, Heart, Left Kidney, Right Kidney, Larynx, Liver, Left Lung, Right Lung, Left Parotid, Right Parotid, Rectum
	Software A (3)	Both Femoral heads, Right submandibular gland
	Software B (2)	Prostate, Right Breast
Quadrant 2 DSC > 0.7 QS < 2	Common (0)	None
	Software A (3)	Prostate, Left Ventricle, Bowel Bag
	Software B (0)	None
Quadrant 3 DSC < 0.7 QS < 2	Common (11)	Bowel, Left Breast, Duodenum, Left Hippocampus, Right Hippocampus, Pelvic Lymph Nodes, Optic Chiasm, Pancreas, Seminal Vesicles, Uterus Cervix, Vagina/Genitalia
	Software A (6)	Left Brachial Plexus, Right Brachial Plexus, Right Breast, Left Cochlea, Lips, Stomach
	Software B (5)	Oral cavity, Right submandibular gland, Constrict Muscles, Left Ventricle, Bowel Bag
Quadrant 4 DSC < 0.7 QS > 2	Common (15)	Left and Right Chest Walls, Trachea, Both Lens, Left Lacrimal Gland, Bronchus, Spinal Cord, Cauda Equina, Pituitary, Both Optic Nerves, Esophagus, Penile Bulb, Right Cochlea
	Software A (2)	Oral cavity, Constrict Muscles
	Software B (7)	Stomach, Lips, both brachial‐plexuses, Left cochlea, both femoral heads

*Note*: The quadrants are divided by DSC = 0.7 and QS = 2 on the QS to DSC scatter plot.

In the second quadrant, there are only two OARs (prostate and left ventricle) from Software A but none from Software B. In the third quadrant, 17 OARs were contoured by Software A but with low quality as assessed by both sets of metrics. In contrast, 16 OARs were delineated by Software B where 11 OARs are in common by both software tools. The challenging structures for both tools are left breast, duodenum, both hippo‐campuses, pancreas, seminal vesicles, vagina/genitalia, bowel bag, optic chiasm, bowel, pelvis lymph nodes, and uterus/cervix. In the fourth quadrant, Software A and Software B contoured 17 and 22 OAR structures well, respectively, as judged by physicians despite low DSCs. Among them, 15 OARs were shared by both tools. These OARs include the left and right chest walls, trachea, both lenses, left lacrimal gland, bronchus, spinal cord, cauda equina, pituitary, both optic nerves, esophagus, penile bulb, and right cochlea. Additionally, Software A generated acceptable contours, according to physician's subjective opinions for the oral cavity and constrictor muscles, while Software B achieved similar performance for seven more OARs which are both brachial plexuses, both femoral heads, stomach, left cochlea, and lips.

## 4 DISCUSSION

This report outlines a framework for radiation oncology facilities to evaluate commercial software and decide on its implementation in clinical practice. The goal is to share our experience on these software's capabilities, challenges, and areas for improvement, thereby providing useful information to other centers for their product selection and clinical adoption of automated contouring solutions. We evaluated the performance of two deep learning‐based auto‐contouring software tools on 58 OAR structures from 36 patients with 7 different disease sites. Two evaluation systems were used: physician's opinions on the clinical usability of the automatically generated contours and quantitative comparison with the contours pre‐approved. This approach aimed to provide independent and objective assessment of the software tools.

The physician's QS, despite being a qualitative metric, is considered more reliable compared to the quantitative metrics like DSC and HD95. One of the major reasons is that there are discrepancies for manual or pre‐drawn contouring traditions, for example, for spinal cord and esophagus, some physicians may have asked to extend over more slices than necessary, which can deteriorate the performance of these quantitative metrics. In addition, small and narrow OAR structures such as brachial plexuses or optic nerves may get over‐punished by quantitative metrics. They are difficult to delineate since they are not easily visible on CT images, but the final contours as shown may actually get approved by physicians. Nevertheless, a correlation study between the physician scores and the quantitative metrics provides an objective assessment of software performance. As shown in Table 4, the contouring results from Software A are more consistent with the physician's opinions compared to those from Software B, although the overall correlation is not that strong in both software tools.

Overall, we found that the two commercial products exhibited comparable performance in terms of contouring accuracy. As displayed in the pie chart of Figure 1, 60% OARs could be well contoured (QS > 2) by both tools and 22% not immediately usable based on the physician's opinions. The scatter plots in Figure 2 also indicate that the two software tools showed similar performance. The average scores for each set of metrics were compared (Table 3), and no significant differences between two tools were observed.

The performance of the software tools for individual OARs, evaluated using both metric systems, is presented in Table 5. The OARs falling in the first quadrant were delineated well (DSC > 0.7 and QS > 2) by both auto‐contouring tools. The common trait of these OARs is their high contrast against surrounding tissues on CT images. In contrast, the OARs in the third quadrant of Table 5, which were contoured poorly by either software, have either inferior contrast on CT images or suffer from unusual anatomical characteristics. For instance, the hippo‐campuses and optic chiasm, embedded within brain soft tissues of similar CT numbers, pose a serious challenge for the auto‐segmentation tool. For these organs, magnetic resonance imaging (MRI), which offers superior soft tissue contrast, is essential for achieving clinically acceptable segmentation accuracy. Meanwhile, ongoing research into MRI‐based synthetic CT auto‐contouring could create additional opportunities for addressing these challenging structures.^13^, ^14^, ^15^ Additionally, OARs with irregular shapes such as the tubular bowels and duodenum, together with those that tend to be affected by significant anatomical variation due to position, motion, and inter‐individual differences, such as breast and pancreas, also posed difficulties for the auto‐contouring tools.

Besides the intrinsic properties with these OARs, another reason for the low‐quality contours from the automatic tools could be the AI models underlying these tools that were not well trained. The sexual organs in female patients with gynecological or pelvic cancers, for example, genitalia, vagina, uterus, etc., could not well delineated with both software tools. This is largely attributed to the insufficient patient data available for the model training, compared to other disease sites,^16^, ^17^, ^18^ which entails further work on the AI model improvement.

There are few OARs that could be reasonably segmented by the tools as listed in the second quadrant of Table 5, indicating again the consistency between the quantitative assessment and physician's opinion. However, a significant number of OARs listed in the fourth quadrant whose contours have low quantitative scores but were clinically acceptable according to physicians. This difference could be due to reasons of soft tissue contrast, their irregular shapes, or the suboptimal AI models, as we described previously. For OARs that do not have ground truths (missing original manual contours) in our evaluation, physician's QS helped recognized them, highlighting the importance of combining both qualitative and quantitative evaluations to obtain a comprehensive assessment.

We would like to acknowledge several limitations in our evaluation process. First, physicians’ opinions are subjective, as they may have personal preference for the generated contours, and physician may treat the OAR delineation differently due to their practice style. Therefore, the QS can only be considered relatively reliable despite the fact that they can be more reliable than the quantitative scores in some scenarios, as we acknowledged. Second, although we aimed to select typical and representative OAR structures, some bias in this selection process is unavoidable, leading to potential systematic errors in the results from both evaluation metrics. Third, the reference contours might not represent the perfect ground truth as explained previously, which could make the quantitative metrics questionable. To address this issue, the reference contours should be manually adjusted so that direct comparison with the automatically delineated contours can be properly made. Fourth, the thresholds to determine the clinical usability in both sets of evaluation systems are arbitrary, especially for the quantitative metrics DSC and HD95, which affects the conclusion on the software performance.

Moreover, the manual contours used as the reference were evaluated based on guidelines from a single institution. Given the variability in contouring practices and adherence to RTOG guidelines across different institutions and physicians, a broader evaluation involving multiple centers and clinicians is warranted to enhance the robustness of the findings. Lastly, according to the dosimetrists involved in this study, ease of use is essential for integrating auto‐contouring tools into the clinical workflow, as it significantly reduces planning time and enhances overall efficiency. In this context, both software tools were user‐friendly and could be well incorporated into the treatment planning process.

Based on our clinical experience at a single institution, we recommend the following practical guide for evaluating auto‐contouring software:

(1) Patient selection: Retrospectively select a number of patients treated across all major sites of the body to include all possible OARs needed in the clinic. Existing contours should adhere to the Radiation Therapy Oncology Group (RTOG) guidelines and have been reviewed by radiation oncologists.
(2) Contour generation: Generate auto‐contours on the same CT scans that include manual contours for direct comparison.
(3) Qualitative evaluation: Have at least two radiation oncologists assess the auto‐contoured structures using a qualitative metric, such as the four‐level scale shown in Table 2.
(4) Quantitative evaluation: Calculate the DSC and HD95 scores based on the quantitative metrics for comparing the automated contours to the manual contours for each of the OARs.
(5) Criteria for adoption: An auto‐contouring software will be recommended to be adopted by a clinic if the automated contours show good accuracy on a good range of OARs involved in the evaluation:
  (a) Good accuracy: High DSC (e.g., ≥0.7) and low HD95, alongside a high QS (e.g., ≥2) as used in this study.
  (b) Broad OAR coverage: High QS for at least 65% of total OARs, with at least two scores meeting a 72% threshold, as demonstrated in this study.

These steps provide a combination of subjective and quantitative evidence to determine whether the auto‐contouring software is suitable for clinical use.

## 5 CONCLUSION

This study provides a comprehensive evaluation of two commercial software tools for automatic contouring based on our single‐institution experience. The software performance was evaluated on the common OAR structures from 36 patients with different cancer types. Both the physicians’ qualitative assessment and the quantitative scores using DSC and HD95 show that either tools can generate clinically acceptable contours for approximately two‐third of the OARs evaluated. However, certain OAR structures remained challenging for both tools due to inferior image contrast, irregular shapes, or the underperforming AI models, etc. We hope that this work provides a roadmap for radiation oncology facilities to evaluate commercial software and make informed decisions about their adoption and integration into clinical practice.

## AUTHOR CONTRIBUTIONS

Tingyu Wang and Ming Chao designed the study and conducted data analysis and interpretation. James Tam, Thomas Chum, Cyril Tai, and Tian Liu also contributed to the data analysis and interpretation. Deborah C. Marshall, Michael Buckstein, Jerry Liu, Sheryl Green, and Robert Stewart provided substantial clinical evaluation of the auto‐contours generated by the AI tools. All authors contributed to the manuscript's development, including drafting, revising, and editing, and have reviewed and approved the final content.

## CONFLICT OF INTEREST STATEMENT

The authors declare no conflicts of interest.

## Data Availability

Research data are stored in an institutional repository and will be available upon request.
